# The Influence of Kapalabhati on Working Memory and Phasic Heart Rate Variability

**DOI:** 10.7759/cureus.61027

**Published:** 2024-05-24

**Authors:** Rana B Budhi, Deepeshwar Singh

**Affiliations:** 1 Division of Yoga and Life Sciences, Swami Vivekananda Yoga Anusandhana Samsthana, Bengaluru, IND; 2 Department of Yoga, Babasaheb Bhimrao Ambedkar University, Lucknow, IND

**Keywords:** high-frequency yoga breathing, cardiac autonomic functions, phasic heart rate variability, working memory, kapalabhati

## Abstract

Background: Cognitive communication abilities, such as working memory (WM), are vital for accomplishing daily activities and are also important for higher-order processes such as planning and problem-solving. The current study investigates the simultaneous effect of kapalabhati (KBH) on WM and phasic heart rate variability (HRV).

Methods: Twenty participants who fulfilled the inclusion and exclusion criteria, with an average age of 23.65±3.07 years (mean±SD), were recruited for the study. Prior to data collection, the participants underwent a seven-day orientation to maintain uniformity in KBH practice. EKGs were assessed using a 16-channel polygraph system arranged in a standard limb lead II configuration. WM was assessed using E-Prime version 2.0 (Psychology Software Tools, Sharpsburg, PA, USA).

Results: There was a significant increase in accuracy after the immediate KBH practice in all three conditions of the WM task (i.e., n-back task: 0-back, 1-back, and 2-back). However, there was also an increase in reaction time. Repeated measures ANOVA of HRV measures showed statistically significant changes in mean rhythm-to-rhythm (RR) intervals, heart rate (HR), number of adjacent N-N intervals over 50 milliseconds (NN50), percentage of successive normal sinus RR intervals greater than 50 milliseconds (pNN50 RR), low frequency (LF), and high frequency (HF), with HR, NN50, pNN50, LF, and HF all significant at p<0.001 and the LF/HF ratio significant at the p<0.01 level.

Conclusion: The results of the current study suggest that KBH practice can modulate vagal tone or parasympathetic activity and improve WM performance. Furthermore, the parasympathetic shift found in the present study may promote better cardioprotective health and longevity.

## Introduction

Orientation, attention, memory, executive functions, and problem-solving are cognitive processes. These abilities are vital for accomplishing daily activities such as paying bills, remembering routes, planning events, remembering lists of things to buy, and remembering appointments [1].

Working memory (WM; n-back task) is one of the cognitive processes; it is a dynamic processing system that has the ability to temporarily store and manipulate information [2]. This ability is crucial for higher-order processes like language comprehension, planning, and problem-solving. According to Oberauer et al., it is also required for complex cognitive activities in daily functions like reasoning, mental arithmetic, reading comprehension, and following instructions [3]. Hansen et al. reported a strong connection between enhanced WM performance and high HRV [4].

Generally, a high HRV indicates good health and a high tolerance for stress resilience, while a low HRV shows a lack of flexibility to respond to changing demands, restricting an individual's ability to respond appropriately to suitable challenges and to resist improper ones. Numerous studies reported that HRV can change under psychological stress [5-7]. Two heart rate variability (HRV) concepts, i.e., tonic and phasic, are used in the experimental design. Tonic HRV corresponds to one-time point resting HRV, while phasic HRV investigates the responsiveness of systems at various time points, like during rest, stress, and recovery [8].

Studies showed that people with a higher tonic HRV perform better on cognitive and executive [9-11] tasks compared to people with a lower tonic HRV [12,13]. However, Britton et al. observed no correlation between HRV and cognitive function [14]. Park et al. reported a strong correlation between a high tonic HRV and an improved phasic HRV [15]. Apart from this, two studies conducted to investigate the relationship between phasic HRV and cognitive performance demonstrated that people with high phasic HRV performed better in cognitive tasks than people with lower phasic HRV [16,17].

Yoga breathing exercises are an important component of yoga practice that emphasizes voluntary regulation of breath [18]. There are several ways in which a practitioner may manipulate their respiration voluntarily; this includes altering the depth, rate, and pace [19]. Kapalabhati (KBH) is a rapid or high-frequency (approx. 1 to 2 Hz) yogic breathing technique that involves active, strong exhalation with passive, effortless inhalation [19]. A recent study investigated the immediate effect of KBH on short-term HRV in healthy participants. This study showed that there was parasympathetic withdrawal immediately after the KBH practice, while after the 20-minute recovery period, there was parasympathetic dominance [20]. The findings suggested that KBH had a parasympathetic shifting effect, and scientific evidence indicates that the parasympathetic shift of HRV promotes good cardiac health and lowers the risk of sudden cardiac death [21]. Previous studies on KBH demonstrated that it enhances immediate memory processing [22], brain capacity [23], lung function [24], and cognitive [25] and physical health [26] on different parameters.

As per the literature review, no study has attempted to investigate the effect of KBH on phasic HRV and WM simultaneously. Henceforth, the present study aims to evaluate the effect of KBH practice at a fixed rate of approximately 1 Hz/sec or 60 strokes/min simultaneously on WM and phasic HRV (i.e., baseline, pre-WM, during KBH practice sessions, and post-WM).

## Materials and methods

Participants

Twenty participants were recruited for the study, with an average age of 23.65±3.07 years. Participants were recruited through convenience sampling and screened using the Montreal Cognitive Assessment [27]; only those who scored 24 or above were considered eligible for the study. The study followed a pre-post experimental design. In the final analysis, data from a total of 16 participants were used; four participants were excluded due to high signal noise and artifacts in the HRV data. The demographic data are shown in Table 1. The study was conducted in the Cognitive Neuroscience Laboratory at Swami Vivekananda Yoga Anusandhana Samsthana in Bangalore, Karnataka, India. Healthy male individuals were recruited from yoga institutions based on advertisements.

**Table 1:** The demographic characteristics of the participants The education level (the majority of the participants were masters; a few were graduates) is shown in the number of years. BMI: body mass index

Variables	Mean±SD
Age	23.65±3.07
Height (m^2^)	1.73±0.04
Weight (Kg)	62.85±9.29
BMI	20.94±3.1
Education	15.25±1.3
Yoga experience	9.81±4.26

Participants with more than six months of yoga experience who were willing to participate in the study and adhere to its protocols were recruited. Females were excluded from this study because some research has found that their autonomic and cognitive functions [28], as well as respiratory variables, can be influenced by the phases of the menstrual cycle [29].

The exclusion criteria for the study were as follows: participants (1) were on any medication that affects cognitive and attention functions; (2) were involved in any other ongoing research activities; (3) had self-reported substance use, including alcohol, tobacco, illicit drugs, or other medicines; (4) had undergone any surgery in the six months prior to the study, such as gastrointestinal surgery; (5) used stimulants, anxiolytics, or antipsychotics; and (6) had cognitive or attention impairments.

A signed informed consent form was obtained from all participants in the study. All study protocols were approved by the Institutional Ethics Committee of Swami Vivekananda Yoga Anusandhana Samsthana (approval number: RES/IEC-SVYASA/110/2017, approval date: December 2, 2017). The trial was registered at the Clinical Trial Registry of India (CTRI/2018/01/011209), and the study was conducted in accordance with the principles of the Declaration of Helsinki.

Data collection

For demographic and anthropometric measures, participants provided basic demographic information (e.g., age, education level, ethnicity, and sex) at baseline. Height (cm) was recorded with a wall-mounted ruler at baseline, and weight (kg) was recorded on a calibrated electronic balance (GTEP Precision Electronic Instruments Model No.11, New Delhi, India) using standard procedures. BMI (kg/m²) was calculated from this information.

Participants were acclimatized to the lab environment before the actual data collection. On the day of data acquisition, they sat in a sound-attenuated, dimly lit cabin with a sound level of 26 dB for the assessments, and the research team monitored them through a glass window in the wall behind a participant to detect if he or she moved during a session. The research team gave instructions through a slight opening in the door, which the team closed immediately after so that participants could remain undisturbed during a session. The recording room temperature was maintained at 24.0±1.0°C. The average humidity was 56% on the days when experiments were conducted. Both autonomic measurements and the results of WM tasks were recorded simultaneously to assess the effects of KBH on HRV and WM.

Autonomic variables

Participants' EKGs were assessed throughout each session, which lasted approximately an hour (i.e., five minutes baseline, 15 minutes pre-WM, 18 minutes KBH sessions, 15 minutes post-WM), using a 16-channel polygraph system (MP 100 BIOPAC, Acknowledge Software, BIOPAC System, Goleta, CA, USA). The team recorded the EKGs using Ag/AgCl pre-gelled electrodes (Tyco Healthcare, Ratingen, Germany), arranged in a standard limb lead II configuration.

The data were acquired at a sampling rate of 1024 Hz. The recorded data were visually inspected in offline mode; only noise-free data were included for analysis. The data included an average of a five-minute epoch for the baseline EKG, a 15-minute epoch for the EKG during the pre-WM task, 18-minute KBH sessions, and 15-minute post-WM. An RR-interval error of 5% was considered acceptable. The team used Kubios HRV version 2.0 (Department of Physics, University of Kuopio, Kuopio, Finland) for the extraction of all the indices of HRV.

**Figure 1:**
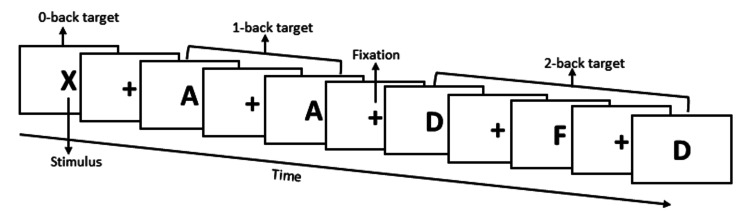
The pattern of stimulus presentation Image Source: Author

The WM experiment was programmed and executed by the research team using E-Prime version 2.0 (Psychology Software Tools, Sharpsburg, PA, USA) [30] on an HP notebook 15-ac101tu laptop (Palo Alto, CA, USA). The E-Studio and E-Data Aid modules within E-Prime were used to design the sequence of the WM task stimuli, assigning diverse capital letters, such as A, B, and C, and using three levels of the WM task: 0-back, 1-back, and 2-back (Figure 1).

The participants were asked to sit comfortably facing the computer screen, and the WM task was explained to them. Before the actual experiment, participants performed practice trials using a different set of stimuli (non-test stimuli) to ensure they comprehended the instructions.

The software presented the items in the test trials involving the testing stimuli at the center of fixation, displaying them as a “+” on the computer screen. Before and after every capital letter (actual stimulus), the fixation “+” appeared on the screen to make the participants more vigilant and prepared for the actual task. The software presented three sets of capital letters at each level, 0-back, 1-back, and 2-back, in a pseudo-random fashion, with 40 stimuli in each set.

The random, capital-letter stimuli were presented serially during the WM tasks, remaining on the screen for 1500 milliseconds with an inter-stimulus interval of 500 ms. The response keypad consisted of four keys arranged in parallel. Participants had to respond by pressing the assigned response key, indicating whether the letter on the screen matched the letter presented X steps back in the 0-back task, one step back in the 1-back task, or two steps back in the 2-back task.

Each participant completed 120 trials at each level, totaling 480 trials per measurement. The frequency of target stimuli was set at 30% of all presented stimuli. The total duration of the tasks was approximately 15 minutes.

Outcome measures

HRV Evaluation

The HRV evaluation included measurements during rest and performance of a WM task: (a) mean rhythm-to-rhythm (RR) interval series, (b) heart rate (HR), (c) standard deviation of RR intervals (STDRR), (d) root mean square of successive differences (RMSSD), (e) triangular interpolation of RR interval histogram (TINN), (f) percentage of successive normal sinus RR intervals >50 milliseconds (pNN50), (g) number of adjacent N-N intervals over 50 milliseconds (NN50), (h) LF, (i) HF, and (j) LF/HF.

WM Task Evaluation

WM was evaluated through accuracy and reaction time in the WM task. Accuracy assessed the correctness of responses, while reaction time measured how quickly a participant responded within the given time frame (in milliseconds).

Statistical analysis

The research team conducted statistical analyses using IBM SPSS Statistics 21.0 software (Armonk, NY: IBM Corp.). The data were normally distributed, and for comparing pre- and post-WM task performance, a paired t-test was conducted. For phasic HRV analysis, since it involved multiple sessions, a repeated measures ANOVA was performed. This involved comparing baseline, pre-WM, KBH1, KBH2, KBH3, and post-WM sessions. Post-hoc analysis was conducted using Bonferroni correction.

Intervention

The KBH experimental group received orientation for seven days, i.e., 18 minutes of KBH practice at 1 Hz (60 strokes per minute or one stroke per second), divided into three sessions of five minutes each with a one-minute break in between.

## Results

In the behavioral outcomes of the WM task, notable patterns emerged. Accuracy showed a marked improvement across 0-back, 1-back, and 2-back conditions immediately after KBH practice, indicating enhanced performance. Conversely, reaction time exhibited a significant increase in mean values for 1-back and 2-back tasks post-KBH practice, suggesting a slower response in these conditions. These findings are detailed in Table 2, presenting mean±SD values. This suggests that the subjects performed more accurately, scoring high.

**Table 2:** Immediate effect of high-frequency breathing on WM performance Note: *p<0.05, **p<0.01, ***p<0.001 compared with baseline or pre-WM WM: working memory

Variable	0-back		1-back		2-back	
	Pre-WM	Post-WM	Pre-WM	Post-WM	Pre-WM	Post-WM
Reaction time (ms)	410.8±31.34	431.08±53.95	403.05±30.4	448.77±61.59**	402.91±34.24	485.08±90.3**
Accuracy (%)	73.57±16.28	90.78±8.4***	49.01±20.32	79.38±11.42***	33.64±13.08	73.44±15.94***

The HRV results obtained during KBH and WM task performance revealed significant findings across several measures. A repeated measures ANOVA analysis indicated statistical significance in mean RR, HR, NN50, pNN50, LF, and HF, all at p<0.001, along with the LF/HF ratio at the p<0.01 level. The detailed ANOVA results are presented in Table 3.

**Table 3:** HRV measures of ANOVA results HF: high frequency, LF: low frequency, RR: rhythm-to-rhythm, STDRR: standard deviation of rhythm-to-rhythm intervals, HR: heart rate, STDHR: standard deviation of heart rate intervals, RMSSD: root mean square of successive differences, NN50: number of adjacent N-N intervals over 50 milliseconds, pNN50: percentage of successive normal sinus rhythm-to-rhythm intervals >50 milliseconds, TINN: triangular interpolation of RR interval histogram, ANOVA: analysis of variance, HRV: heart rate variability

Domain	Measures	Factor	F(df) = value	p-value	Partial eta square
Frequency domain analysis	HF	States	F(4.57,54.84) = 5.99	p<0.001	η2 = 0.33
	LF	States	F(4.58,54.96) = 6.15	p<0.001	η2 = 0.34
	LF/HF ratio	States	F(2.1,25.14) = 10.08	p<0.01	η2 = 0.46
Time domain analysis	Mean RR	States	F(2.16,25.9) = 15.2	p<0.001	η2 = 0.56
	STDRR	States	F(3.15,37.83) = 0.9	NS	η2 = 0.07
	Mean HR	States	F(2,23.96) = 14.98	p<0.001	η2 = 0.56
	STDHR	States	F(3.48,41.74) = 0.48	NS	η2 = 0.04
	RMSSD	States	F(3.04,36.47) = 2.28	NS	η2 = 0.16
	NN50	States	F(1.68,20.12) = 22.02	p<0.001	η2 = 0.65
	pNN50	States	F(2.45,29.38) = 13.08	p<0.001	η2 = 0.52
	TINN	States	F(3.64,43.67) = 1.89	NS	η2 = 0.14

Bonferroni correction, conducted for post-hoc analysis, revealed that in frequency domain analysis during the second session of KBH, there was a statistically significant decrease in HF and an increase in LF, indicating sympathetic arousal at p<0.01. Additionally, there was a significant increase in the LF/HF ratio during the first and second sessions of KBH.

In time domain analysis, there was a significant decrease in mean RR in all three sessions at p<0.001, p<0.01, and p<0.01, respectively. Similarly, there was a decrease in NN50 and pNN50 as well. Conversely, mean HR increased statistically significantly during all three sessions of KBH at p<0.001, p<0.01, and p<0.05 in the first, second, and third sessions of KBH, respectively. All the mean±SD values are displayed in Table 4.

**Table 4:** HR variability result for baseline, during KBH practice, and WM task Note: *p<0.05, **p<0.01, ***p<0.001 compared with baseline or pre-WM. ^$^p<0.05, ^$$^p<0.01 compared with pre-WM HF: high frequency, LF: low frequency, RR: rhythm-to-rhythm, STDRR: standard deviation of rhythm-to-rhythm intervals, HR: heart rate, STDHR: standard deviation of heart rate intervals, RMSSD: root mean square of successive differences, NN50: number of adjacent N-N intervals over 50 milliseconds, pNN50: percentage of successive normal sinus rhythm-to-rhythm intervals >50 milliseconds, TINN: triangular interpolation of RR interval histogram, BS: baseline, WM: working memory, KBH: kapalabhati

Variables	BS	Pre-WM	KBH1	KBH2	KBH3	Post-WM
HF	47.78±16.41	46.2±13.1	26.47±23.86	20.1±19.72**	29.82±21.75	44.38±12.07
LF	52.13±16.48	53.41±12.99	73.45±23.95	79.84±19.76**	70.09±21.81	55.29±12.15
LF/HF	1.51±1.49	1.41±1.03	6.42±5.44*	7.76±6.04*	4.78±4.72	1.42±0.69
Mean RR (ms)	868.37±106.43	827.51±103.54	731.22±79.78***	762.49±84.97**	774.73±92.88**	849.25±101.45$
STDRR (ms)	71.99±33.12	75.98±32.62	57.44±16.64	67.51±63.21	66.78±40.57	78.03±27.63
Mean HR (1/min)	70.67±9.16	74.32±10.43	83.47±8.85 ***	80.39±8.48**	79.04±8.82*	72.32±9.72$$
STDHR (1/min)	5.85±2.93	6.31±2.06	6.67±2.92	6.7±5.21	5.96±3.2	6.33±1.86
RMSSD (ms)	63.99±35.31	72.64±45.13	37.64±34.31	48.67±73.1	51.22±52.8	71.28±41.26
NN50 (count)	109.85±88.09	305.23±192.19	12±10.15*	18.62±28.41*	33.31±43.04	253.54±193.39
pNN50	29.06±22.49	26.17±17.39	2.89±2.55*	4.87±8.09*	8.81±11.72*	25.32±20
TINN (ms)	270.77±123.47	374.62±108.21	342.31±142.78	303.46±158.47	290.77±135.54	351.15±105.58

Post-hoc analysis with Bonferroni adjustment during pre- and post-WM of KBH practice revealed an increase in mean RR values during the post-WM task after the KBH practice at p<0.05. In contrast, there was a decrease in mean HR during the WM task after the immediate KBH practice at p<0.01. The mean±SD values have been reported in Table 4. In other variables of HRV, there were no statistically significant changes.

## Discussion

In the current study, we investigated phasic HRV during baseline (rest), pre-WM (psychological stress (PS)), during KBH session practice (PS), and post-WM (PS). To our knowledge, this is the first pilot study to simultaneously investigate the immediate effect of KBH practice on both phasic HRV and WM performance.

The study results revealed that KBH at approximately 1 Hz/sec (60 strokes/min) for 15 minutes, with a one-minute break after every five-minute session, increased sympathetic nervous system activity during practice and improved mental processes during post-WM performance. The findings of the current study show a significant decrease in HF and an increase in LF and the LF/HF ratio power during KBH practice in frequency domain analysis, indicating sympathetic arousal. Similarly, time-domain analysis of HRV measures showed a decrease in mean RR, NN50, and pNN50 and an increase in mean HR, which also indicates sympathetic arousal during KBH practice. The changes in HRV indices during KBH practice are similar to those observed with physical exercise, as reported in previous studies [31]. However, all HRV measures returned to normal immediately after practice. Our findings align with a previous study [32]; additionally, our study reports a significant increase in mean HR during KBH practice. Though this may be an exaggeration, the findings suggest that older adults or physically disabled individuals may receive benefits similar to mild physical exercise by practicing KBH.

Furthermore, there was a statistically significant increase in mean RR during post-WM task performance compared to pre-WM after the KBH practice. In contrast, there was a decrease in mean HR during the post-WM task after the immediate KBH practice compared to pre-WM. This suggests that after the KBH practice, the participants were able to perform the WM task comfortably and also scored high in accuracy. This indicates that the KBH practice helped the participants to remain alert in a rest-like state in the presence of post-psychological stress (WM task).

Previously, studies reported that active inhalation improves facial expression recognition and object recall [33]. KBH practice comprises vigorous exhalation and passive inhalation through the nose at a faster rate; this breathing technique may also directly affect recognition and recall [34]. Another earlier study reported that the immediate effect of KBH improved visual perception [35]. In the current study, the aforementioned ability may have partially accounted for the improvement in accuracy in WM task performance after the KBH practice.

In terms of neurophysiology, successful social behavior adaptation depends on the withdrawal and re-establishment of parasympathetic activity, which represents a supporting mechanism for the metabolic requirements that alter in response to environmental challenges [36,37]. A decreasing phasic HRV, indicated by its indices HF, LF, and LF/HF ratio (frequency domain) and mean RR, NN50, pNN50, and mean HR (time domain), during physiological stress (i.e., KBH) like physical exercise, such as in the current study, indicates parasympathetic withdrawal and triggers a defensive system to manage it [38]. Additionally, studies have shown that changes in HRV are associated with changes in cerebral blood flow in brain regions crucial for self-regulation, such as executive function, emotional regulation, stress management, social function, and inhibitory processes [39,40]. Experiments show that HRV is linked to self-regulation efforts [15]. Self-regulation helps the body mobilize physiological resources and enables goal-directed behavior in response to shifting demands [2]. Both increasing and decreasing HRV reactivity appear to be adaptive in response to the demands of a situation [13,36]. The findings of the present study suggest that the activation of self-regulatory mechanisms could account for the pattern of sympathetic activation coupled with parasympathetic withdrawal during KBH. Evidence suggests that the use of self-regulation resources is associated with higher parasympathetic withdrawal, indicating an adaptive function [15,39] when people are exposed to physiological or psychological stressors [8] to mobilize and deliver energy for the body to handle the stressor [36]. In the current study of phasic HRV, it was found that during pre-WM and KBH, there was parasympathetic withdrawal, and during post-WM after KBH practice, parasympathetic activity was re-established, and the participants' accuracy improved. The findings of this study are consistent with a previous study, which found that KBH practice has a parasympathetic shifting effect [20].

The generalizability of the current study's findings is constrained by the small sample size, the absence of a control group, and the inclusion of only healthy male subjects. A future study with a larger sample size, a randomized control group, and both male and female participants is required to scientifically validate the findings of the present study. Additionally, the future study should consider the cut-off level for the accuracy of the WM task, include a post-event session (i.e., after post-WM), and involve simultaneous recording of respiration to authenticate the findings of the present study.

## Conclusions

The KBH is a yogic breathing practice that involves active and forceful exhalation using abdominal muscle contractions, followed by natural and passive inhalation. Therefore, KBH can be energizing, cleansing (removing carbon dioxide), and heating (during KBH practice, sympathetic arousal and parasympathetic withdrawal occur). Furthermore, the findings of the present study indicate that KBH practice can modulate the vagal tone or parasympathetic activity and improve WM performance. Hence, the parasympathetic shifting effect of KBH, as found in this study, may be cardioprotective, promoting better health and longevity.
